# Integrating deep learning and regression models for accurate prediction of groundwater fluoride contamination in old city in Bitlis province, Eastern Anatolia Region, Türkiye

**DOI:** 10.1007/s11356-024-34194-w

**Published:** 2024-07-11

**Authors:** Ayşegül Demir Yetiş, Nagehan İlhan, Hatice Kara

**Affiliations:** 1 Medical Services and Techniques Department, Bitlis Eren University, 13000 Bitlis, Türkiye; 2 Department of Computer Engineering, Harran University, 63050 Şanlıurfa, Türkiye; 3 GAP Agriculture Research Institute, 63100 Şanlıurfa, Türkiye

**Keywords:** Groundwater, Heavy metal contamination, Prediction, Fluoride, Deep learning, Türkiye

## Abstract

Groundwater resources in Bitlis province and its surroundings in Türkiye’s Eastern Anatolia Region are pivotal for drinking water, yet they face a significant threat from fluoride contamination, compounded by the region’s volcanic rock structure. To address this concern, fluoride levels were meticulously measured at 30 points in June 2019 dry period and September 2019 rainy period. Despite the accuracy of present measurement techniques, their time-consuming nature renders them economically unviable. Therefore, this study aims to assess the distribution of probable geogenic contamination of groundwater and develop a robust prediction model by analyzing the relationship between predictive variables and target contaminants. In this pursuit, various machine learning techniques and regression models, including Linear Regression, Random Forest, Decision Tree, K-Neighbors, and XGBoost, as well as deep learning models such as ANN, DNN, CNN, and LSTM, were employed. Elements such as aluminum (Al), boron (B), cadmium (Cd), cobalt (Co), chromium (Cr), copper (Cu), iron (Fe), manganese (Mn), nickel (Ni), phosphorus (Pb), lead (Pb), and zinc (Zn) were utilized as features to predict fluoride levels. The SelectKbest feature selection method was used to improve the accuracy of the prediction model. This method identifies important features in the dataset for different values of *k* and increases model efficiency. The models were able to produce more accurate predictions by selecting the most important variables. The findings highlight the superior performance of the XGBoost regressor and CNN in predicting groundwater quality, with XGBoost consistently outperforming other models, exhibiting the lowest values for evaluation metrics like mean squared error (MSE), mean absolute error (MAE), and root mean squared error (RMSE) across different *k* values. For instance, when considering all features, XGBoost attained an MSE of 0.07, an MAE of 0.22, an RMSE of 0.27, a MAPE of 9.25%, and an NSE of 0.75. Conversely, the Decision Tree regressor consistently displayed inferior performance, with its maximum MSE reaching 0.11 (*k* = 5) and maximum RMSE of 0.33 (*k* = 5). Furthermore, feature selection analysis revealed the consistent significance of boron (B) and cadmium (Cd) across all datasets, underscoring their pivotal roles in groundwater contamination. Notably, in the machine learning framework evaluation, the XGBoost regressor excelled in modeling both the “all” and “rainy season” datasets, while the convolutional neural network (CNN) outperformed in the “dry season” dataset. This study emphasizes the potential of XGBoost regressor and CNN for accurate groundwater quality prediction and recommends their utilization, while acknowledging the limitations of the Decision Tree Regressor.

## Introduction

Water has been considered an inexhaustible resource until recently when water scarcity due to the decrease in its quality and quantity has become a major problem (Yurtseven et al. 2010; Selek and Demir Yetiş 2017). The depletion of available surface water resources due to pollution and decreased precipitation in recent years has increased the need for groundwater use. Groundwater resources are widely used for drinking water supply because they are of better quality than surface waters and are sufficient in quantity based on the population of the settlement (Klove et al. 2017; Demir Yetiş et al. 2018). Regular routine monitoring is required to ensure the suitability of groundwater quality for drinking water. Otherwise, it is likely to pose serious risks and hazards to public health. Groundwaters are known as water bodies that are rapidly exposed to pollution by anthropogenic and geogenic factors despite being of higher quality than surface waters (Egbueri and Agbasi 2022).

Groundwater is the preferred water source for domestic, industrial, and agricultural activities (Narsimha et al. 2012; Derin et al. 2020; Bayhan et al. 2020). Therefore, the quality of water and its suitability for various uses are of importance to human and environmental health. The natural chemical quality of waters in the sublayers of the soil is generally suitable for all types of use. However, as the water moves downward from the surface, it comes into contact with organic matter, soil, and rocks in the layers for a certain period. Consequently, changes in groundwater quality may occur, causing some problems due to high concentrations of certain components (Nagaraju et al. 2016; Selek and Demir Yetiş 2017). Hence, understanding the quality and hydrogeochemical properties of groundwater and determining its suitability for various uses is crucial (Brindha and Elango 2011). Settlement areas, domestic, commercial, industrial, and agricultural activities are among the factors that affect groundwater quality, and regular monitoring of groundwater is of great importance (Jassas and Merkel 2015). Therefore, knowing the details of polluting sources and continuing follow-up monitoring in this regard are important steps that must be considered for the protection of water resources. Thus, the effects will create serious pressures on both groundwater and surface water resources, resulting in environmental pollution. Pollutant sources are caused by anthropogenic and geogenic factors. Anthropogenic sources are divided into two categories: point sources and non-point sources. The most well-known types of point source pollution sources are domestic and industrial wastewater treatment plants and solid waste disposal areas. Non-point source pollution sources include agricultural activities, livestock, atmospheric transport, land use, non-point discharges, and open dumping of solid waste areas (Selek and Demir Yetiş 2017). Increasing human population and economic development have led to the accumulation of heavy metals in water bodies due to the transport of pollution sources discussed in Water Resources. This has led to a deterioration of water quality worldwide. Among the pollutants in aquatic ecosystems, heavy metals are the most persistent due to their slow degradation under natural conditions (Varol 2013).

Heavy metals in aquatic environments originate from natural and anthropogenic sources such as atmospheric deposition, geological weathering, agricultural activities, and residential and industrial products. In urban environments, humans are exposed to heavy metals through food, beverages, and water. The accumulation of metal pollutants such as Cd, Cr, Ni, and Pb can lead to growth retardation, kidney diseases, cancer, and many other adverse health effects, particularly in children (Saeedi et al. 2012). Metal ions such as lead, zinc, copper, cobalt, cadmium, chromium, nickel, arsenic, fluoride, mercury, and silver are important both for living organisms and environmental health due to their persistent effects. When these metal ions exceed certain limits, they can have extremely toxic effects, along with certain ailments (Gadd and Griffits 1977, Wong and Kwok 1992, Saglam and Cihangir 1995).

Fluorine (F) is one of the essential trace elements (Aghapour et al. 2018). Fluorine is present in groundwater, which can be highly variable, depending on the structure of rocks and the formation of minerals containing fluoride. According to the International Chemical Safety Program (2002) (IPCS 2002), the World Health Organization (WHO) has classified fluoride as one of the 10 most important chemical substances for public health (Antonijevic et al. 2016). High concentrations of fluoride ions in groundwater are known to affect more than 260 million people worldwide in varying degrees of health. Therefore, many researchers have attached great importance to determining the factors controlling the distribution of fluoride in groundwater (Hamzaoui-Azaza et al. 2011, 2012). The fluoride concentration in different water sources is mainly dependent on the nature of rocks and other minerals that make up the soil. Additionally, some physicochemical factors such as pH, temperature, total dissolved solids, alkalinity, porosity, acidity of the parent material, and well depth can affect the fluoride concentration in groundwater (Aslani et al. 2019). Because of the accumulation of fluoride in the human body, particularly with prolonged exposure to drinking water, it can lead to adverse health effects. The majority of fluoride that enters the body stems from water and correlates with the hydrogeochemistry of water fluoride (Raju 2017).

Inadequate or excessive intake of fluoride can have adverse effects on children’s health. When the concentration of fluoride in drinking water is less than < 0.5 mg/L, there is a risk of dental caries (Aslani et al. 2019). Studies have demonstrated that fluoride can reduce tooth decay by up to 40% (Guissouma et al. 2017). Studies have shown that excessive intake of fluoride from drinking water can cause fluorosis, which negatively affects teeth and bones (Podgorski and Berg 2022). The World Health Organization (WHO) has stipulated a minimum fluoride concentration of 1.5 mg/L (WHO 2022; Demir Yetiş et al. 2021; Prasad and Vithanage 2023) as a guideline. More than 200 million individuals across various developed and developing nations consume drinking water with fluoride concentrations exceeding the standard guidelines (Emenike et al. 2018). Consequently, this has prompted health departments to revise screening guidelines for safe levels of fluoride concentrations in drinking water and provide safe benchmark values, as high fluoride exposure is linked to health-related diagnoses (Ding et al. 2011). In this context, the US Department of Health and Human Services (US-HHS) has revised the existing guidelines for fluoride concentration in drinking water from the range of 0.7–1.2 mg/L to 0.7 mg/L. This updated recommendation is grounded on scientific assessments from both US-EPA (US Environmental Protection Agency) and US-HHS, with the primary aim of minimizing tooth decay and mitigating potential health risks. Water containing fluoride concentration above this limit can lead to various diseases such as dental fluorosis and skeletal fluorosis (Fordyce et al. 2007; Amini et al. 2016). Fluorosis, previously regarded as solely a dental issue, has now developed into a significant health concern, causing ailments such as joint and muscle pain. Skeletal fluorosis in its early stages is characterized by symptoms of discomfort and stiffness in the spine, hip area, and joints. Additionally, excessive fluoride results in the calcification and ossification of spinal ligaments (Raju 2017). In addition, high concentrations of fluoride in water can result in various health issues, including damage to the nervous system, reduced fertility, impaired intellectual development in children, urinary tract diseases, IQ reduction in children, and hypertension, as well as adverse effects on neurological functions, Alzheimer’s disease, and cancer (Kaoud and Kalifa 2010; Razdan et al. 2017; Emenike et al. 2018; Derin et al. 2023). Excessive fluoride in drinking water, dietary intake, water consumption, nutrient intake, climatic factors, and duration of exposure may impede fluoride assimilation in the human body, leading to detrimental effects. Consequently, the impact of comparable fluoride levels may vary among different populations, contingent on their immune competence (Raju 2017).

Fluoride analysis in drinking water is usually done using chemical methods, which provide accurate and reliable results. However, these methods can be costly, labor-intensive, and time-consuming, leading to the development of alternative techniques for determining certain parameters without actually measuring them. As a result, various techniques have been developed to provide easier and faster results There are many studies worldwide that investigate the amount of fluoride in drinking water and its effects on human health (Raju et al. 2017; Emenike et al. 2018; Yousefi et al. 2019; Aravinthasamy et al. 2020; Adeyeye et al. 2021; Chicas et al. 2022; Yazıcı Karabulut et al. 2023).

In the study proposed, we identify the potential distribution of geogenic contaminated groundwater (hydrogeological and geochemical conditions) and build a strong model to predict the contamination by establishing relationship between predictive elements with the key contaminant, fluoride. Knowledge of fluoride development is crucial in successfully managing and combating fluoride-related epidemiological issues (Raju 2017). Groundwater resources are a crucial freshwater source for the historical city of Bitlis and its surroundings, particularly in the Eastern Anatolia region of Türkiye (Kul 2021). This water source primarily serves drinking and domestic purposes in the region, with additional use for irrigation. As such, consistent monitoring of the water quality is imperative to ensure the health and well-being of those who rely on it. However, the groundwater utilized for drinking in the area has high levels of fluoride present in certain locations. The highest levels of fluoride detected in the groundwater sources situated in and around the Bitlis province measured 2.17 mg/L during the rainy season and 3.26 mg/L during the arid period (Yilman 2022). For planning and management purposes, it is crucial to possess data on groundwater containing elevated levels of fluoride. It is also essential to determine and approximate the specific fluoride values. In this scope, fluoride was estimated, and the measurement of heavy metal parameters in the study was made exclusively in the ICP device, without chemicals. For the first time, we estimated the level of fluoride contamination in groundwater using several regression algorithms, traditional models, and deep learning techniques based on values of toxic metals. The majority of the models achieved a prediction accuracy with lower error rates. There have been very few recent studies on predicting groundwater quality using these techniques (Barzegar et al. 2017; Yeşilnacar et al. 2008; Cao et al. 2022; Podgorski and Berg 2022), and there have been no studies that specifically rely on heavy metal parameter measurements.

## Material and method

### Study area

The total area of Bitlis province, located within the boundaries of the Eastern Anatolia Region, is 6707 km^2^. It encompasses a part of 1876 km^2^ of Lake Van, making the overall surface area of the province 8645 km^2^. It is situated between 41°33′ and 43°11′ east longitudes and 37°54′ and 38°58′ north latitudes. Bitlis has seven districts: Bitlis Center, Adilcevaz, Ahlat, Güroymak, Hizan, Mutki, and Tatvan. The elevation of the province is 1545 m above sea level (PESR 2022). Due to its high altitude from sea level, Bitlis has a continental climate. The winters are cold and snowy, while the summers are hot and dry. The temperatures in the province range from an average of − 19 to 36.8 °C. The annual precipitation is 958 mm. Agriculture and animal husbandry are the main livelihood sources in Bitlis province. Due to the mountainous, rocky, and forested terrain, agricultural areas are limited. Only 18.9% of the city’s land is agricultural land. Due to the rugged topography, small-scale and large-scale animal husbandry is predominantly practiced in the province (PESR 2022; Kul 2021; Yilman 2022).

### Geological settings

The province of Bitlis and the district of Guroymak, which occupy a large area in the northern part of the Tigris River Basin, are studied in the lower Tigris sub-basin. The districts of Tatvan and Ahlat, located in the north of the Van Closed Basin, are examined within the boundaries of the Van Closed Basin. In the northern part of the Lower Tigris Basin, the Bitlis Metamorphics, which cover a large area and represent the oldest unit (Precambrian-Devonian), consist of metamorphic bedrock formations such as metagabbro, metarhyolite (metavolcanics), serpentine, amphibolite, gneiss, schist, phyllite, recrystallized limestone, marble, quartzite, pelagic limestone, and radiolarite (DSI-General Directorate of State Hydraulic Works 2015; Kul 2021; Yilman 2022). The Tatvan sub-basin, located north of the Van Closed Basin, is composed of metamorphic, igneous, and sedimentary units. Because of volcanism occurring in Mount Nemrut, the basin’s western part (Tatvan-Bitlis road location) consists of volcanic units, while the eastern part is composed of sedimentary units. The Ahlat sub-basin also includes both igneous and sedimentary units. Due to the volcanism in Mount Nemrut, the western, northern, and southern parts of the basin are formed by volcanic units, while the eastern part shows the distribution of sedimentary units. In the Adilcevaz sub-basin, there are both igneous and sedimentary units. As a result of volcanism occurring in Mount Suphan, located near the northeastern border of the basin, the eastern and northeastern parts of the basin are composed of volcanic units. The sedimentary units are distributed in the southern and western parts of the basin (DSI-General Directorate of State Hydraulic Works 2013; Kul 2021; Yilman 2022).

The discharge measurements of the units belonging to the Bitlis Metamorphic range from 0.1 to 1.0 L/s, and they have a low aquifer characteristic. Precipitation waters in the region drain into the outlets of streams, rivers, or low-discharge springs in the valley bottoms, depending on the topographic elevations. The Quaternary alluvial unit that surfaces in the Tatvan aquifer area is not considered as an aquifer due to its thin layer overlying the Quaternary volcanic unit and its limited distribution in a small area. Similarly, the Quaternary alluvial units that surface in the Ahlat aquifer area are not considered an aquifer due to their thin layer overlying the Lower Pliocene–Quaternary volcanic units. Based on the data obtained from the drilled and existing wells on the aquifer, it is observed that the total thickness of the aquifer varies between at least 150–200 m. Upon examining the wells, it is observed that the volcanic units continue until the end of the well, and there is no entry into the impermeable bedrock. However, considering the geological structure of the basin, it is estimated that beneath the volcanic units forming the aquifer area, there are Neogene (20 million years ago) aged marl units. In the Ahlat sub-basin, the aquifer exhibits the characteristics of a free aquifer. The Adilcevaz sub-basin constitutes an aquifer area consisting of Quaternary alluvium, Pliocene–Quaternary volcanic units, and the underlying Neogene-aged limestone units. The alluvial unit forming the flat areas of the aquifer generally consists of clay, silt, sand, and gravel materials, with a thickness of 142 m. As one moves to higher elevations, volcanic units are found beneath the lower part of the alluvial unit. In the study conducted in the Adilcevaz aquifer, it is observed that the impermeable bedrock of the aquifer is composed of Neogene-aged impermeable clayey marl units (DSI-General Directorate of State Hydraulic Works 2013; Kul 2021; Yilman 2022).

### Field and sampling studies

Water samples were collected from Bitlis and its districts to determine the levels of heavy metals in the water sources and groundwater. Sampling was conducted during the rainy period of June 18–25, 2019, and the dry period of September 17–18, 2019. A different feature of Bitlis province is that it has two important critical periods. The months of May and June, which we call the rainy period, represent the period when the snow that fell until April begins to melt and the rain begins. The dry period represents the end of the dry summer season and the period just before the autumn rains begin.

**Fig. 1:**
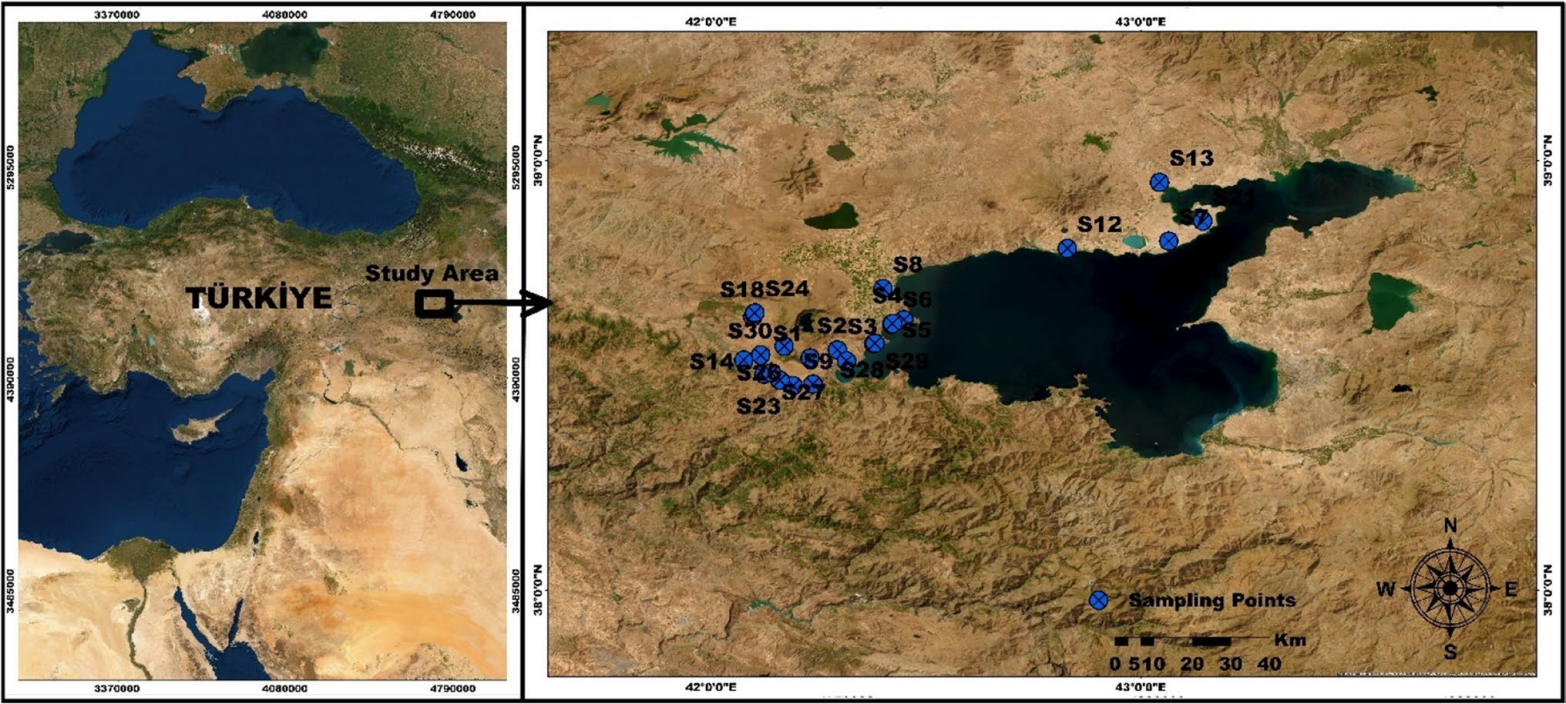
Location map and sampling points of the study area (Yilman 2022)

The water samples from the study area were collected by the guidelines “TS ISO 5667–11 Water Quality- Sampling- Part 11: Guide for Sampling Groundwater” and “TS 9359 Water Quality-Sampling from Monitoring Wells for Groundwater-Guidelines” and under the conditions specified in APHA storage guidelines. Table 1 and Fig. 1 show the coordinates and locations of the sampling points in the study region. In the field, characteristics such as pH, temperature, and conductivity were determined in groundwater samples. The ICP-OES (Perkin Elmer 7000) equipment was used to quantify heavy metals in three duplicates. The fluoride parameter was measured using a UV spectrometer (APHA-AWWA-WEF, 2012). Ultra-pure water was used to create the solutions and standards for the analyses. Every chemical utilized was of the highest analytical grade and additional purity (Merck). Certified reference materials (UME CRM 1201, spring water) were utilized to assure the correctness of the analyses.

**Table 1:** Location information and coordinates of sampling points

Sampling points	Locations	Elevation (m)	Coordinate	
			X (Latitude)	Y (Longitude)
S1	Benekli 1	1859	38°32′20″	42°13′53″
S2	Yumurtatepe 1	1881	38°33′32″	42°17′35″
S3	Yumurtatepe 2	1879	38°33′32″	42°17′37″
S4	Adabağ 1	1675	38°37′40″	42°26′23″
S5	Sarıkum 1	1659	38°37′03″	42°25′24″
S6	Sarıkum 2	1679	38°37′14″	42°25′19″
S7	Kavuştuk 1	1666	38°48′45″	43°03′54″
S8	Saka 1	1689	38°42′03″	42°23′57″
S9	Küllüce 1	1819	38°28′36″	42°11′26″
S10	Gölbaşı 1	1332	38°38′20″	42°05′54″
S11	Gölbaşı 2	1331	38°38′34″	42°05′56″
S12	Yolçatı 1	1735	38°47′45″	42°49′44″
S13	Heybeli 1	1710	38°56′58″	43°02′36″
S14	Kekliktepe 1	1607	38°32′09″	42°04′35″
S15	Örenlik 1	1871	38°28′49″	42°14′12″
S16	Yazıkonak 1	1837	38°30′13″	42°07′21″
S17	Yazıkonak 2	1831	38°30′12″	42°07′21″
S18	Gölbaşı 3	1330	38°38′40″	42°06′03″
S19	Toki1	1683	38°31′59″	42°18′54″
S20	Toki 2	1686	38°32′00″	42°18′53″
S21	Yarımada 1	1654	38°51′36″	43°08′42″
S22	Koruk 1	1808	38°29′20″	42°09′35″
S23	Koruk 2	1808	38°29′22″	42°09′36″
S24	Gölbaşı 4	1336	38°38′38″	42°06′16″
S25	Oduncular 1	1882	38°34′05″	42°10′12″
S26	Oduncular 2	1883	38°34′05″	42°10′12″
S27	Tahtalı Köyü	1806	38°30′26″	42°08′25″
S28	Kıyıdüzü 1	1677	38°34′25″	42°22′40″
S29	Kıyıdüzü 2	1662	38°34′28″	42°22′50″
S30	Aşağı kolbaşı	1639	38°32′52″	42°06′53″

### Dataset

In the rainy season dataset, there are 27 data points, while the dry season dataset comprises 29 data points, resulting in a total of 56 data points in the all dataset. The dataset consists of aluminum (Al), boron (B), cadmium (Cd), cobalt (Co), chromium (Cr), copper (Cu), iron (Fe), manganese (Mn), nickel (Ni), phosphorus (Pb), lead (Pb), zinc (Zn), and fluoride (F) features. Statistical measures of the datasets are given in Table 2. Initially, the data was divided into a training set (70%), a validation set (15%), and a test set (15%). The model was trained on the training set, and its performance was assessed on the validation set, with accuracy scores calculated for both sets. Subsequently, the training accuracy was compared with the validation accuracy to identify potential overfitting, which, upon testing, showed no evidence thereof. Following the overfitting assessment, the dataset was partitioned into 70% for training and 30% for testing.

**Table 2:** Statistical measures of the datasets

Dry season dataset													
Metrics	Al	B	Cd	Co	Cr	Cu	Fe	Mn	Ni	P	Pb	Zn	F
count	29.00	29.00	29.00	29.00	29.00	29.00	29.00	29.00	29.00	29.00	29.00	29.00	29.00
mean	0.05	0.13	0.04	0.13	0.11	0.19	0.15	0.03	0.00	0.14	0.05	0.00	0.33
std	0.20	0.21	0.16	0.06	0.16	0.20	0.21	0.11	0.03	0.21	0.09	0.00	0.25
min	0.00	0.00	0.00	0.03	0.00	0.00	0.00	0.00	0.00	0.00	0.00	0.00	0.03
0.25	0.00	0.01	0.00	0.12	0.01	0.03	0.00	0.00	0.00	0.03	0.00	0.00	0.14
0.50	0.00	0.05	0.00	0.12	0.01	0.09	0.05	0.00	0.00	0.07	0.00	0.00	0.28
0.75	0.00	0.21	0.00	0.12	0.27	0.41	0.26	0.01	0.00	0.14	0.06	0.00	0.45
max	1.00	1.00	0.87	0.32	0.44	0.58	1.00	0.48	0.14	0.95	0.28	0.00	1.00
Wet season dataset													
Metrics	Al	B	Cd	Co	Cr	Cu	Fe	Mn	Ni	P	Pb	Zn	F
count	27.00	27.00	27.00	27.00	27.00	27.00	27.00	27.00	27.00	27.00	27.00	27.00	27.00
mean	0.04	0.14	0.05	0.21	0.22	0.10	0.11	0.09	0.04	0.15	0.30	0.04	0.32
std	0.13	0.23	0.19	0.24	0.25	0.21	0.21	0.26	0.19	0.19	0.35	0.19	0.20
min	0.00	0.00	0.00	0.00	0.01	0.00	0.00	0.00	0.00	0.01	0.00	0.00	0.00
0.25	0.00	0.01	0.00	0.12	0.01	0.00	0.00	0.00	0.00	0.05	0.00	0.00	0.17
0.50	0.00	0.05	0.00	0.12	0.05	0.01	0.01	0.00	0.00	0.12	0.20	0.00	0.34
0.75	0.00	0.20	0.00	0.12	0.39	0.09	0.16	0.02	0.00	0.16	0.67	0.00	0.50
max	0.65	0.84	1.00	1.00	1.00	1.00	1.00	1.00	1.00	1.00	1.00	1.00	0.66
All dataset													
Metrics	Al	B	Cd	Co	Cr	Cu	Fe	Mn	Ni	P	Pb	Zn	F
count	56.00	56.00	56.00	56.00	56.00	56.00	56.00	56.00	56.00	56.00	56.00	56.00	56.00
mean	0.05	0.14	0.04	0.17	0.17	0.15	0.13	0.06	0.02	0.14	0.17	0.02	0.33
std	0.17	0.21	0.17	0.18	0.21	0.21	0.21	0.20	0.13	0.20	0.28	0.13	0.23
min	0.00	0.00	0.00	0.00	0.00	0.00	0.00	0.00	0.00	0.00	0.00	0.00	0.00
0.25	0.00	0.01	0.00	0.12	0.01	0.00	0.00	0.00	0.00	0.04	0.00	0.00	0.16
0.50	0.00	0.05	0.00	0.12	0.01	0.05	0.04	0.00	0.00	0.10	0.00	0.00	0.30
0.75	0.00	0.21	0.00	0.12	0.35	0.23	0.20	0.02	0.00	0.15	0.24	0.00	0.49
max	1.00	1.00	1.00	1.00	1.00	1.00	1.00	1.00	1.00	1.00	1.00	1.00	1.00

### Methodology

#### Evaluation metrics

Evaluation metrics play an important role in assessing the performance and effectiveness of machine learning and predictive models. They provide quantifiable metrics that help researchers, data scientists, and practitioners understand how well their models are performing and whether they are achieving the desired goals. In this section, we discuss the evaluation metrics used in this study: mean squared error (MSE), mean absolute error (MAE), root mean squared error (RMSE), Nash–Sutcliffe efficiency (NSE), and mean absolute percentage error (MAPE).

The main contributions of the study are as follows:

- **Utilization of regression models**: To build a model representing the relation between geogenic variables and fluoride levels, various regression methods such as Linear Regression, Random Forest, Decision Tree, K-Neighbors, and XGBoost were applied. These techniques helped to uncover the link between predictor variables and the target pollutant fluoride.
- **Utilization of deep learning techniques**: In addition to regression approaches, deep learning techniques such as artificial neural networks (ANN), deep neural networks (DNN), convolutional neural networks (CNN), and long short-term memory (LSTM) have been used. These techniques are recognized for their ability to capture complex patterns and correlations in data, hence improve model prediction skills.
- **Using SelectKbest feature selection:** The SelectKbest feature selection method was used to improve the accuracy of the prediction model. This method identifies important features in the dataset for different values of *k* and increases model efficiency. The models were able to produce more accurate predictions by selecting the most important variables.
- **Detailed seasonal analysis**: A detailed seasonal analysis was conducted to assess the potential impacts of seasons on groundwater pollution. Geogenic variables and fluoride levels were grouped according to dry and wet seasons and seasonal changes were analyzed.
- **Evaluation metrics**: Metrics such as mean squared error (MSE), mean absolute error (MAE), and root mean squared error (RMSE) were used to assess the prediction models’ performance. These metrics provided a quantitative measure of prediction accuracy and allowed different models to be compared. The majority of the models achieved high predictive accuracy with low error rates, demonstrating their ability to predict geologically contaminated groundwater.

## Mean squared error (MSE)

MSE is a fundamental metric used to measure the average squared difference between the actual and predicted values in a regression problem. It is calculated by taking the average of the squared differences between the predicted (${\widehat{y}}_{i}$) and actual values (${y}_{i}$).

$$MSE=\frac{1}{n}\sum_{i=1}^{n}{\left({\widehat{y}}_{i}-{y}_{i}\right)}^{2}$$

The MSE is advantageous because it penalizes large errors more than small ones, making it particularly sensitive to outliers. However, it is not expressed in the original units of the data, making interpretation difficult.

## Mean absolute error (MAE)

MAE is another metric commonly used in regression tasks. It calculates the average absolute difference between predicted and observed values:

$$MAE=\frac{1}{n}\sum_{i=1}^{n}{\left|{\widehat{y}}_{i}-{y}_{i}\right|}^{2}$$

Unlike MSE, MAE is less sensitive to outliers as it does not square the errors. MAE is expressed in the same units as the original data, making it easier to interpret. It provides a clearer indication of the average magnitude of error in the predictions.

## Root mean squared error (RMSE)

RMSE is derived from MSE by taking the square root of the mean squared differences between the predicted and observed values:

$$RMSE=\sqrt{\frac{1}{n}\sum_{i=1}^{n}{\left({\widehat{y}}_{i}-{y}_{i}\right)}^{2}}$$

RMSE shares many features with MSE but is more interpretable because it is in the same units as the target variable. Like MSE, RMSE is sensitive to outliers but penalizes large errors more severely due to the square root operation.

## Nash–Sutcliffe efficiency (NSE)

NSE is a performance measure widely used in hydrology and environmental modeling to assess the predictive ability of a model. It measures the relative magnitude of the residual variance compared to the variance of the observed data. NSE is calculated as follows:

$$NSE=1-\frac{\sum_{i=1}^{n}{\left({y}_{i-}{\widehat{y}}_{i}\right)}^{2}}{\sum_{i=1}^{n}{\left({y}_{i-}\overline{y }\right)}^{2}}$$

where ${y}_{i}$ is the observed value at time *i*, ${\widehat{y}}_{i}$ is the predicted value at time *i*, $\overline{y }$ is the mean of the observed values and *n* is the number of observations. A value of 1 for NSE indicates a perfect fit, while values less than 0 indicate that the mean of the observed data is a better predictor than the model. NSE values between 0 and 1 indicate the model's predictive ability with respect to the mean of the observed data.

## Mean absolute percentage error (MAPE)

MAPE is a widely used metric for evaluating forecasting models, especially in time series analysis. It calculates the average percentage difference between the predicted and observed values relative to the observed values.

$$MAPE=\frac{1}{n}\sum_{i=1}^{n}\frac{\left|{y}_{i-}{\widehat{y}}_{i}\right|}{{y}_{i}} x 100$$

The MAPE metric helps to interpret and compare different data sets by showing the accuracy of the model’s predictions as a percentage.

## Feature selection

The process of selecting features is an essential stage in both machine learning and statistical modeling, with the main objective of identifying and utilizing the most pertinent features to construct precise and effective predictive models. The SelectKBest method is a well-known strategy for feature selection, especially for datasets with a large number of features (Darst et al. 2018). It involves ranking features based on their statistical importance in relation to the target variable and choosing the top *k* features. The initial step of the process involves individually evaluating each feature using a designated scoring function. This scoring function utilizes statistical tests like ANOVA *F*-value, chi-squared statistic, or mutual information to determine the relevance of each feature to the target variable. A higher score indicates a greater predictive significance of the feature for the target variable. Once all features have been scored, SelectKBest selects the top *k* features with the highest scores and disregards the remaining ones. The value of *k* can be determined through domain expertise, experimentation, or automated methods like cross-validation.

## Framework

**Fig. 2:**
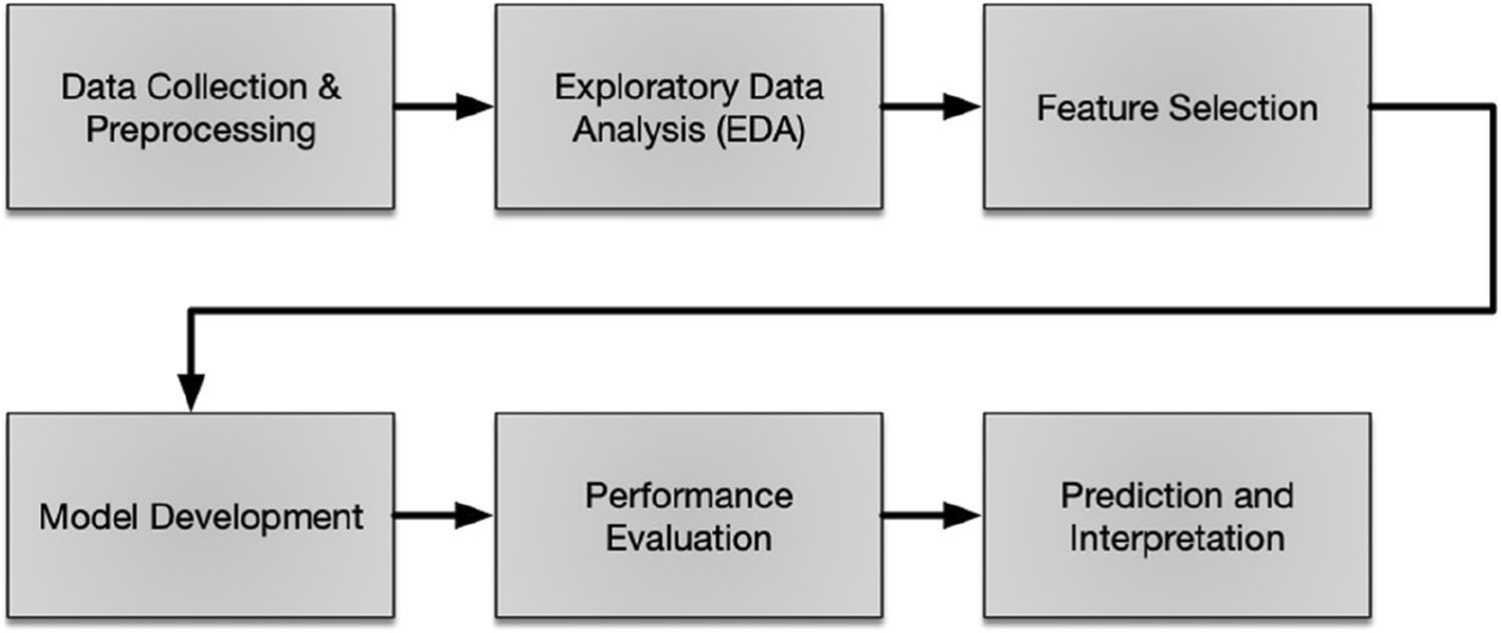
Stages of the framework

This section proposes a framework for predicting fluoride concentrations in groundwater. The framework consists of collecting and preprocessing data, exploratory data analysis, feature selection, developing models using regression and deep learning techniques, performance evaluation, and prediction and interpretation stages. Access to safe, clean drinking water is vital for human health worldwide, as groundwater serves as the primary source. However, groundwater is vulnerable to contamination from natural factors, including elements such as aluminum (Al), boron (B), cadmium (Cd), cobalt (Co), chromium (Cr), copper (Cu), iron (Fe), manganese (Mn), nickel (Ni), phosphorus (Pb), lead (Pb), and zinc (Zn). The presence of these variables can significantly impact water quality. The framework aims to examine how geogenic variables influence fluoride levels, which are especially concerning in developing nations where mitigation is challenging and dental and other health issues related to both insufficient and excessive fluoride are common. Monitoring fluoride levels holds great importance because of potential health issues at high amounts. To estimate fluoride level in groundwater, we propose a framework as shown in Fig. 2.

The proposed comprehensive framework is established to predict fluoride concentration in groundwater, enabling informed decision-making and interventions to ensure the safety of drinking water sources. Each step performed in each stage is listed sequentially.

1. Data collection and preprocessing:
  Collect groundwater samples from diverse locations, aiming for a balanced representation across a range of geological and hydrogeological settings.
  Assess the levels of specific elements within the collected groundwater samples, including Al, B, Cd, Co, Cr, Cu, Fe, Mn, Ni, P, Pb, and Zn, as well as the focal parameter, F (fluoride).
  Perform necessary data preprocessing steps such as handling missing values, outlier detection and treatment, and data normalization. Execute essential data preprocessing procedures such as addressing missing data, identifying and addressing anomalies, and data normalization.
2. Exploratory data analysis (EDA)
  Perform preliminary dataset analysis to acquire an understanding of the distribution and interconnections among the attributes and the objective variable.
  Compute concise statistical summaries, create graphical representations of distributions, and detect potential trends or associations through the utilization of scatter diagrams, histograms, correlation matrices, etc.
3. Feature selection
  Conduct feature selection to determine the most pertinent features for estimating the target variable F (fluoride).
  Implement a feature selection method suitable for regression, such as SelectKBest, which eradicates features based on their significance or coefficients obtained from a regression model.
  Use the subset of selected features for additional analysis and model development.
4. Model development
  To assess the models’ performance, divide the dataset into training and testing subsets.
  Select from several regression algorithms, including Linear Regression, Random Forest, Decision Tree, K-Neighbors, and XGBoost, for model creation.
  Using the chosen features obtained from the feature selection procedure, train each regression model with the training set.
  The distribution of geogenic contaminated groundwater can be predicted using deep learning techniques, specifically artificial neural networks (ANN), deep neural networks (DNN), convolutional neural networks (CNN), and long short-term memory (LSTM) in addition to traditional regression models. The deep learning models should be trained and assessed using the same datasets employed for the traditional regression modeling.
  The deep learning models should be trained and assessed using the same datasets employed for the traditional regression modeling.
  The performance of each model should be evaluated using suitable evaluation metrics, including MSE, MAE, RMSE, NSE, and MAPE.
  A comparison between the performance of traditional regression models and deep learning models should be carried out.
5. Performance evaluation
  Compare the performance of all developed models based on evaluation metrics.
  Assess their accuracy and error rates to determine their predictive capability for the target variable F (fluoride).
  Select the best-performing model(s) based on the evaluation results.
6. Prediction and interpretation
  Employ the best-performing model(s) to forecast the fluoride concentration (F) in groundwater samples from novel/unfamiliar locations.
  Analyze the predictions and examine the correlations between the designated features and the anticipated fluoride concentration to obtain an understanding of the factors influencing water quality.

It should be noted that the implementation details and parameter tuning for each regression algorithm and deep learning technique may differ. The methodology provided above offers a general framework for feature selection and building regression models to forecast the fluoride concentration in groundwater.

## Results and discussion

### Data visualization and distribution analysis

Data visualization is employed in this section to gain insights into the distribution characteristics of the variables under investigation. Boxplot distributions for the features, which include aluminum (Al), boron (B), cadmium (Cd), cobalt (Co), chromium (Cr), copper (Cu), iron (Fe), manganese (Mn), nickel (Ni), phosphorus (P), lead (Pb), zinc (Zn), and fluorine (F), are presented to facilitate a comprehensive understanding. A valuable tool for summarizing data distributions, revealing central tendencies, and identifying potential outliers within the dataset are boxplots. The boxplots are generated from three distinct datasets, namely the “all dataset,” “rainy season,” and “dry season,” allowing for a comparative analysis of these seasons’ environmental attributes.

**Fig. 3:**
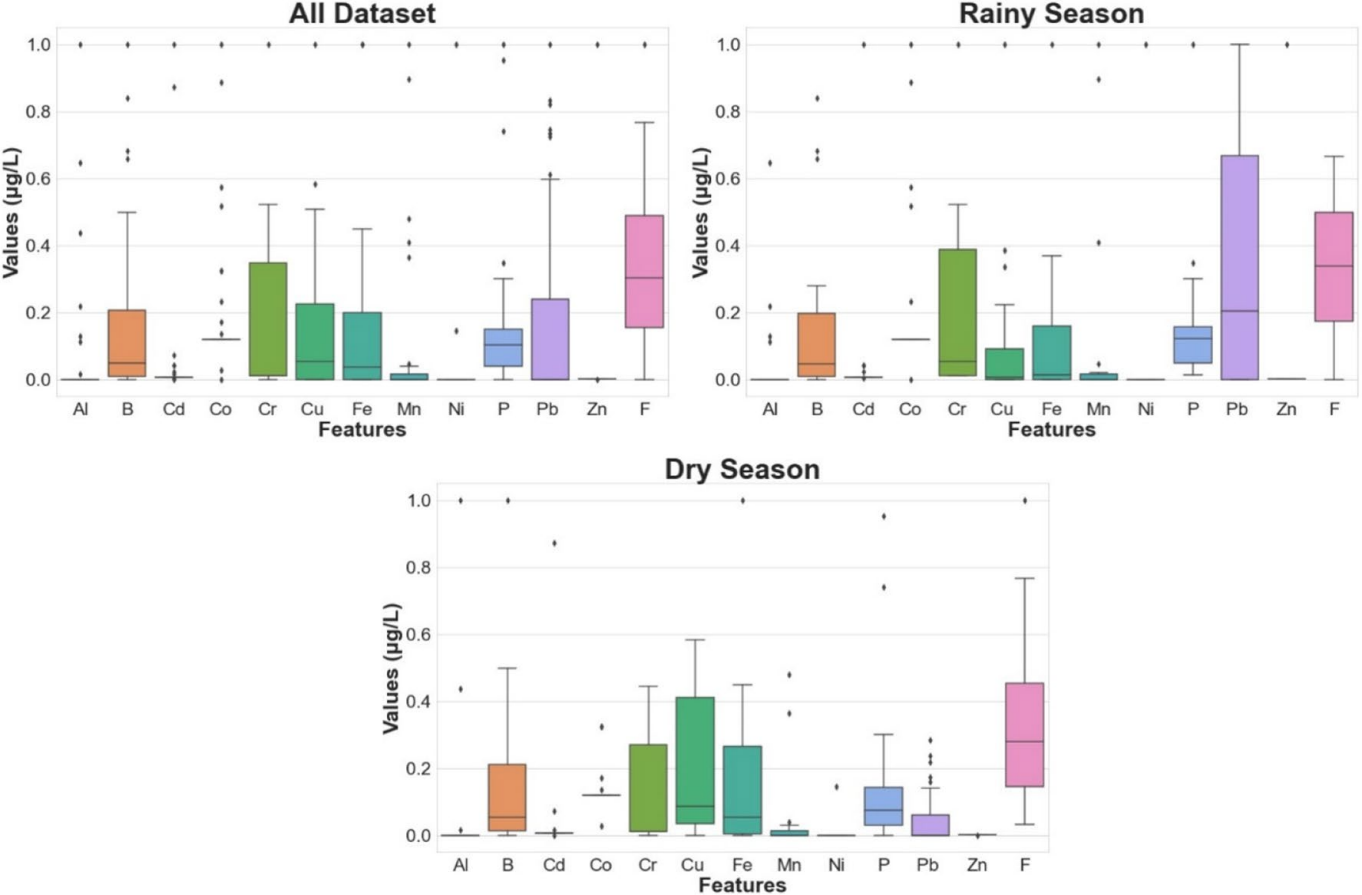
Feature correlations

The box plots in Fig. 3 are used to show how the datasets are distributed in different seasons (Selek and Demir Yetiş 2017). The all dataset appears to have a wide range of values across many columns. Some features such as Al, Cd, Co, and Mn in “All dataset” exhibit a significant number of outliers, indicating the presence of extreme values. The redox transformation process, dissolution of schistose rock and sulfide minerals can be seen as the source of Cd value in the groundwater system, while Co is resulted from coal combustion, cobalt alloys, and industrial effluents. In the study, Al and Mn values are affected by rock weathering, mineral dissolution, water–rock interaction, ore processing, domestic waste, and agricultural activities. There is variation in the spread of values across different columns. In rainy season, some features, like Cu, Pb, and Zn, have a concentration of values around the median, with few outliers. Overall, rainy season appears to have a more compact distribution compared to all dataset. In dry season, several columns, such as B, Cu, Fe, P, and Zn, have a wider spread of values and a significant number of outliers. Like rainy season and dry season generally, it has a more compact distribution compared to “All dataset.” Fig. 3 shows that the dominant and prominent parameters in the dataset of three periods are B, Cr, Pb, and F. The fact that the region is an old region contains volcanic and geothermal resources and geogenic factors can be shown as the reason for this situation (Karabulut et al. 2023; Derin et al. 2023).

### Feature correlations

In this part, we provide the correlation analysis of 12 elements (Al, B, Cd, Co, Cr, Cu, Fe, Mn, Ni, P, Pb, and Zn) and one factor (F) in our dataset which covers both rainy and dry seasons. The correlation analysis measures the strength and direction of the linear relationship between each pair of elements or factors. The results are presented in a correlation matrix, where each cell contains the correlation coefficient between two elements or factors. The correlation coefficient varies between − 1 and 1, where − 1 signifies a complete negative linear relationship, 0 signifies no linear relationship, and 1 signifies a complete positive linear relationship. The results reveal the correlation patterns between items in the dataset by recognizing the pairs with the strongest or weakest linear relationship.

**Fig. 4:**
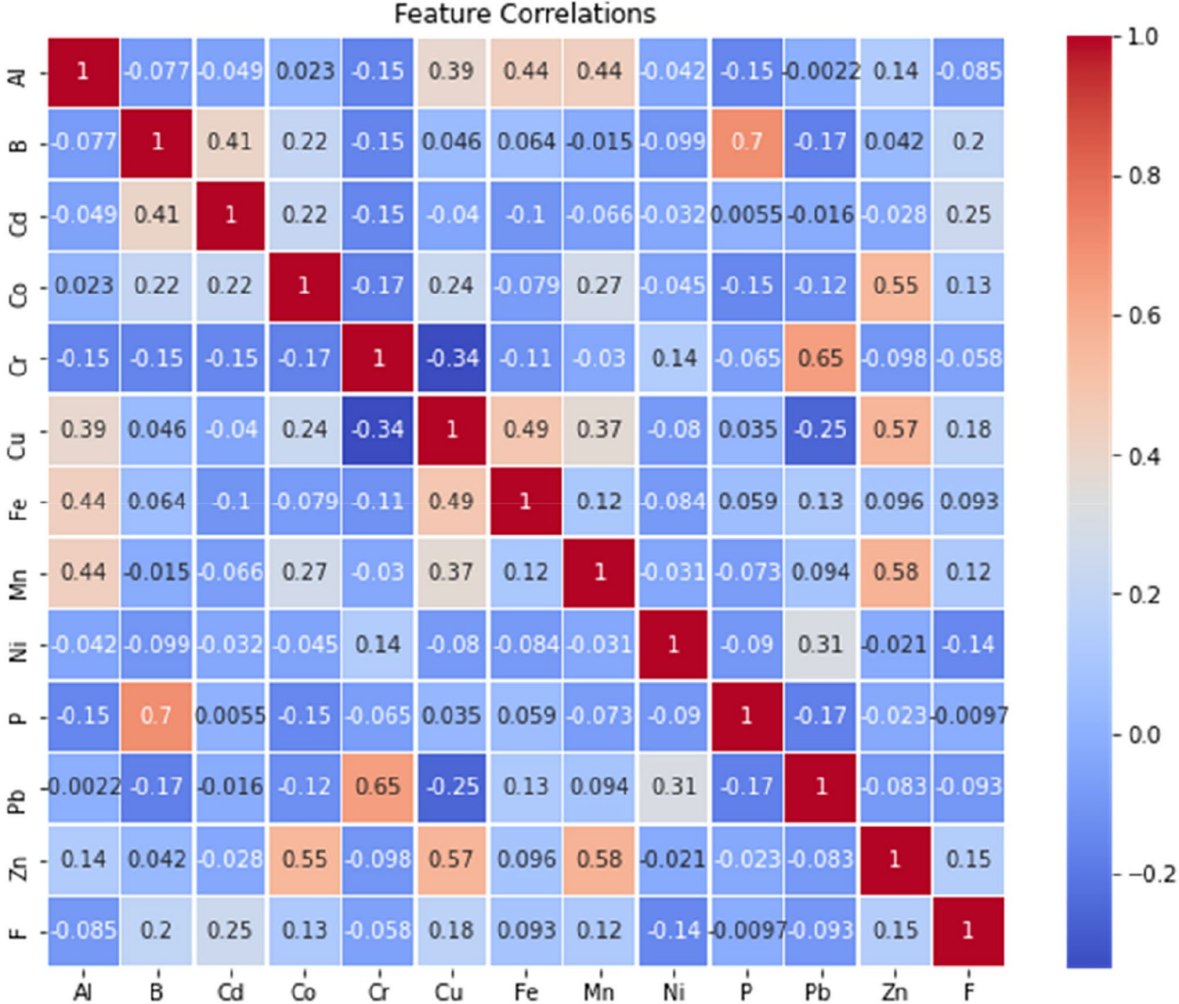
Feature correlations

Figure 4 shows the results of the correlation matrix. The strongest and positive linear connection in the matrix is between B and P with a correlation coefficient of 0.79. This shows that as B increases, P also tends to increase. The lowest negative correlation coefficient of the matrix is − 0.34 and is between Cr and Cu. This means that Cr and Cu have a moderate negative linear relationship, meaning that Cu tends to decrease when Cr increases. The highest absolute correlation coefficient in the matrix is 0.70, between B and P. This means that B and P have the strongest linear relationship among all pairs of elements or factors in the dataset. The lowest absolute correlation coefficient in the matrix is 0.093, between Fe and F. This means that Fe and F have the weakest linear relationship among all pairs of elements or factors in the dataset. Some pairs of elements or factors have a correlation coefficient close to zero, such as Cd and P (0.0055), Al and Pb (− 0.0022), and B and Fe (0.064). This means that these pairs have no or very weak linear relationships. Some pairs of elements or factors have a correlation coefficient close to one or negative one, such as B and P (0.70), Cr and Pb (0.65, Co and Zn (0.55), and Al and Cu (0.39). This means that these pairs have a strong or very strong linear relationship. Upon examining the correlations between the toxic elements affecting fluoride, it was found that there is a strong positive correlation (0.25–0.2) between fluoride, Cd, and B. Additionally, technical abbreviations have been explained upon first use. Geogenic and anthropogenic inputs are mostly responsible for the enrichment of B and F content in groundwater. Since some parts of the study area have a semi-arid environment with basaltic hard rock terrain, it has Ca + Mg-HCO_3_ as the main water type that promotes the dissolution of F and B in groundwater. In addition, the reasons that increase the B and F content are inorganic fertilizers used in agricultural areas and domestic wastewater (Kadam et al. 2020). Meanwhile, the lowest correlation with fluoride was observed between P, Fe, Al, Cr, and Pb. Therefore, it can be concluded that Cd and B show a better correlation with fluoride than the other elements evaluated. Furthermore, it is evident that the rock structure found in extensive geological and geographical layers serves as the primary source of all components responsible for fluoride contamination in groundwater (Bera and Ghosh 2019; Chowdhury et al. 2019; Araya et al. 2022). Additionally, the interaction between water and rocks is among the geogenic factors that contribute to the prolonged retention and gradual movement of water containing fluoride (Raju 2017). Battaleb-Looie (2012) reported that calcite, limestone, and intertwined marl were the sources of high fluoride in a similar area (Ling et al. 2022).

### Feature selection results

**Fig. 5:**
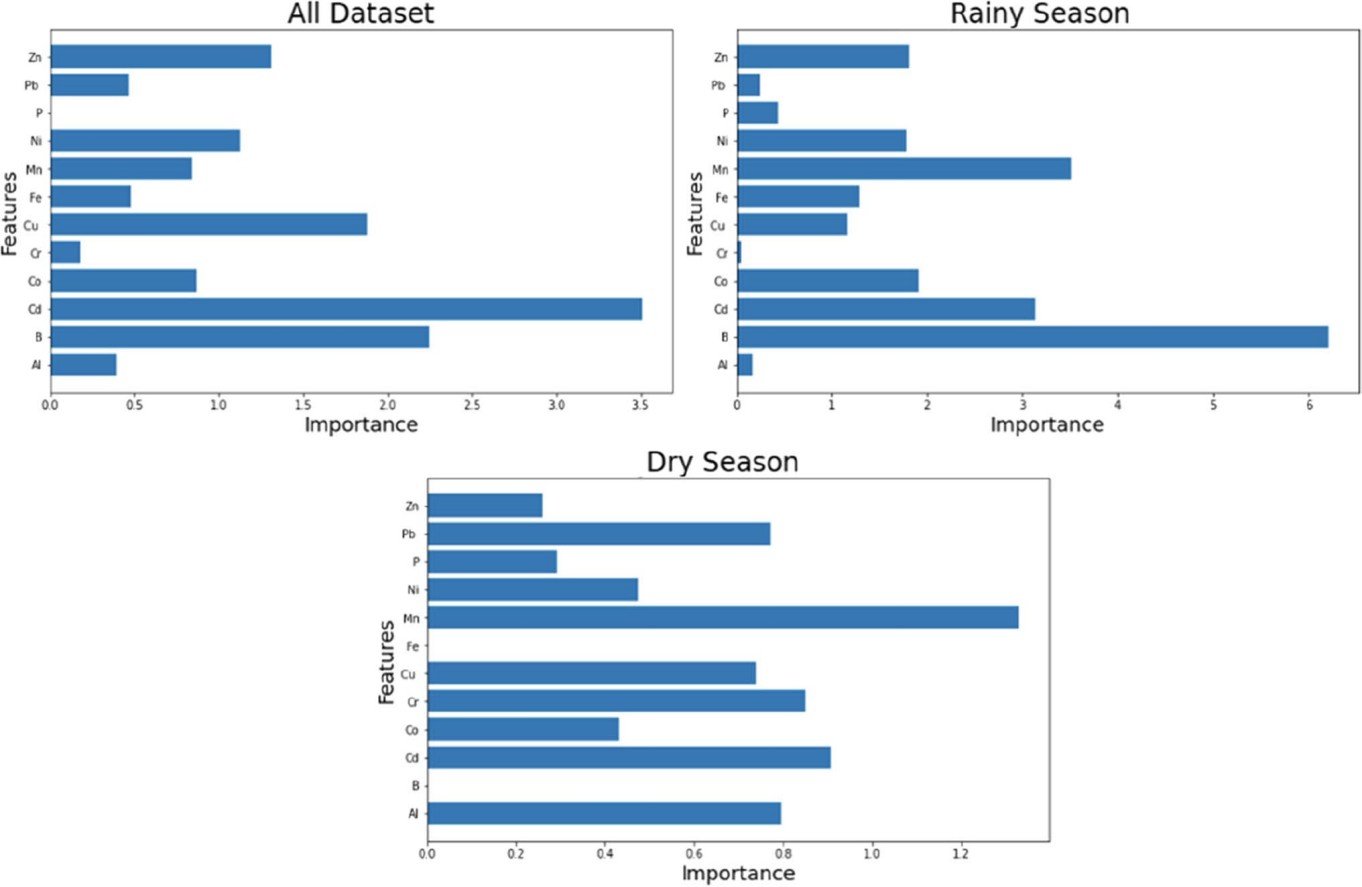
Feature importances

Figure 5 shows the feature importances of 12 elements (Al, B, Cd, Co, Cr, Cu, Fe, Mn, Ni, P, Pb, and Zn) in three datasets: “all,” the “rainy season,” and the “dry season” datasets. The feature importances were calculated using SelectKBest feature selection method. The figure reveals the differences in feature importance among the three datasets for the 12 elements and also indicates the most and least important features in each dataset and the seasonal variations in feature importance.

The most important feature in all datasets is Cd, with a score of 3.508. The results of Cd concentrations of the present study were lower than the maximum value measured in Khan et al.’s 2016 study. The least important feature is P, with a score of 0.005. The most important feature in the rainy season dataset is *B*, with a score of 6.207. *B* values are mostly affected by natural sources, including local geological conditions, volcanic activity, and hydrothermal processes (Rodriguez-Espinosa et al. 2020). The least important feature is Cr, with a score of 0.049. The most important feature in the dry season dataset is Mn, with a score of 1.331. The least important feature is Fe, with a score of 0.0001. The occurrence of Cd and Mn in groundwater may be due to the interaction of groundwater with mafic and ultramafic rocks, agricultural activities and industrial wastewater. Additionally, weathering and dissolution of schistose rocks and sulfide minerals in the study area may contaminate the groundwater aquifer with Cd.. Dissolution of granitic rocks also increases Cd values in groundwater.. B has the highest difference in feature importance between rainy and dry seasons, with a decrease of 6.206 in the dry season. Fe has the lowest difference, with a decrease of 0.001 in the dry season. Cd, Co, Cu, Mn, Ni, and Pb have higher feature importance in the dry season than in the rainy season. Al, Cr, Fe, P, and Zn have lower feature importance in the dry season than in the rainy season. The average feature importance in all datasets is 1.18. The average feature importance the in rainy season dataset is 1.94. The average feature importance in dry season dataset is 0.62. The rainy season dataset has higher feature importance than all dataset for B, Cd, Co, Mn, and Ni. The all dataset has higher feature importance than the rainy season dataset for Al, Cr, Cu, Fe, P, Pb, and Zn. The dry season dataset has higher feature importance than the all dataset for Cd and Mn. The all dataset has higher feature importance than the dry season dataset for Al, B, Co, Cr, Cu, Fe, Ni, P, Pb, and Zn.

Table 3 shows the selected features for three datasets (all dataset, rainy season dataset, and dry season dataset) using select best feature selection method with different values of *k* (9, 7, 5, and 3). The features with the highest importance are selected for each dataset. B and Cd are the only features chosen across all datasets and values of *k*, indicating their indispensable role in influencing the factor F. Co, Cu, Mn, Ni, and Zn are present in datasets for *k* = *9* and *k* = *7*, but not for *k* = *5* and *k* = *3*. This indicates that these characteristics are significant but not indispensable in the dataset and their impact on factor *F* varies depending on the value of *k*. Al, Cr, Fe, P, and Pb are included in certain datasets but not in others, as well as only for certain values of *k*. This suggests that these characteristics have less significance or relevance within the dataset, with a seasonal or conditional impact on factor *F*. It is noteworthy that Al is the sole characteristic chosen exclusively in the dry season dataset, as opposed to the rainy season dataset or the complete dataset, indicating that Al’s impact on factor *F* is specific to the dry season only. P is the only feature that is selected in rainy season dataset but not in dry season dataset or all dataset. This reveals that P has a specific influence on the factor *F* in rainy season only. Fe is the only feature that is selected in rainy season dataset and all dataset but not in dry season dataset. This demonstrates that Fe has a general influence on the factor *F* but not in dry season. Several studies conducted on groundwater in and around Bitlis have reported high levels of iron and manganese ratio. The presence of Fe in groundwater is attributed to ionic activity and gneiss rock formation (Emenike et al. 2018). Cr and Pb are the only features that are selected in dry season dataset and all dataset but not in rainy season dataset. This reflects that Cr and Pb have a general influence on the factor *F* but not in rainy season. With the dissolution of chromium and mica minerals in volcanic rocks, Al, Fe, and P can pass into water with fluoride. Generally, due to the low intensity of human activity in terms of industrial and residential areas within the study area, fluoride in groundwater cannot have an anthropogenic origin. Therefore, a high concentration of fluoride in groundwater is geogenic, meaning that it is influenced by local hydrogeological conditions (Yeşilnacar et al. 2016). Fluoride is a highly prevalent trace element found in the Earth’s crust, with an average concentration of 625 mg/kg in various rock types (Edmunds and Smedley 2005). Some of these rocks may also contain fluoride-rich accessory minerals. However, the mere existence of biotite alone is enough to generate dissolved fluoride levels exceeding 4 mg/L (Raju 2017). The primary sources of fluoride in groundwater are fluoride-bearing minerals found in the Earth’s crust, such as fluorite, fluorapatite, micas, amphiboles, cryolite, villiaumite, topaz, and certain clays. These minerals are the most prevalent fluoride-bearing minerals in natural environments (Hem 1985). The role of pH is crucial in hydrogeological conditions, with ion exchange processes promoting fluoride leaching under alkaline pH conditions (Ling et al. 2022). Groundwater in the research area, which comprises granite gneissic rock, showed alkalinity with elevated pH values (Kul 2021). Fluoride concentration in the groundwater increases in an alkaline environment, as a result of the high apatite and biotite solubility and the desorption of fluoride ions from minerals containing fluoride (Raju 2017). The rate of fluoride dissolution may be higher in sodium-bicarbonate waters as found by Kul (2021) in the study area. Furthermore, the release of fluoride from clay minerals is highly dependent on pH (Saxena and Ahmed 2003). The high level of fluoride in groundwater is impacted not only by geogenic conditions in the region but also by dry weather conditions (climate), the alkaline soil environment and the distribution of clay. In regions of the world with arid climates or seasons, high temperatures prevail, causing water levels to decrease and minerals to become more concentrated. This effect of arid climate influences the fluoride concentration in water, as aquifer recharge is affected (Handa 1975; Smedley et al. 2002; Lü et al. 2016; Ling et al. 2022; Araya et al. 2022).

**Table 3:** Selected features

	All dataset	Rainy season dataset	Dry season dataset
*k* = *9*	B, Cd, Co, Cu, Fe, Mn, Ni, Pb, Zn	B, Cd, Co, Cu, Fe, Mn, Ni, P, Zn	Al, Cd, Co, Cr, Cu, Mn, Ni, P, Pb
*k* = *7*	B, Cd, Co, Cu, Mn, Ni, Zn	B, Cd, Co, Fe, Mn, Ni, Zn	Al, Cd, Cr, Cu, Mn, Ni, Pb
*k* = *5*	B, Cd, Cu, Ni, Zn	B, Cd, Co, Mn, Zn	Al, Cd, Cr, Mn, Pb
*k* = *3*	B, Cd, Cu	B, Cd, Mn	Cd, Cr, Mn

### Framework results

The following tables compares the performance of different regression and deep learning models on the datasets (“all dataset,” “rainy season,” “dry season”) with different values of *k*, which is a parameter that controls the number of features used for prediction. The examination of model effectiveness for different *k* values reveals how changing the number of nearest neighbors affects the accuracy of the model. The tables use five evaluation metrics: mean squared error (MSE), mean absolute error (MAE), root mean squared error (RMSE), mean absolute percentage error (MAPE), and Nash–Sutcliffe efficiency (NSE). Lower values of the first four metrics indicate better performance, while higher values of NSE indicate better performance (Barzegar et al. 2017).

Table 4 represents the results of all dataset. Both the Random Forest regressor and XGBoost regressor have consistently displayed impressive performance across all values of *k* (such as *k* = 9, *k* = 7, and *k* = 5), with minimal error metrics (MAE, MSE, RMSE, MAPE) and high NSE values. These models have proven to be robust and effective in capturing complex patterns within the data, regardless of the chosen *k* value. In comparison, the Decision Tree regressor performs adequately but tends to have slightly higher error metrics than the Random Forest regressor and XGBoost regressor, especially at higher *k* values. The K-Neighbors regressor shows varying performance depending on the chosen *k* value, with fluctuating error metrics. However, it generally falls behind the Random Forest regressor and XGBoost regressor in terms of overall performance. LSTM consistently outperforms other deep learning models for all values of *k*, exhibiting lower error metrics and higher NSE values. This indicates that the inclusion of temporal dependencies makes LSTM particularly effective in situations with varying *k* values. In contrast, ANN, DNN, and CNN demonstrate acceptable performance, but generally have higher error metrics than LSTM across different *k* values. However, the performance gap between LSTM and these models is not as significant as that between Random Forest regressor/XGBoost regressor and other regression models. This analysis emphasizes the significance of considering the selection of *k* when choosing a regression or deep learning model. While Random Forest regressor and XGBoost regressor consistently perform well for different *k* values, LSTM stands out as the top performer among deep learning models, particularly in scenarios with varying *k* values. These findings can assist in model selection based on the specific characteristics of the dataset and the prediction requirements. It is worth mentioning that, in all *k* values, Random Forest regressor and XGBoost regressor consistently exhibit better performance than other regression models. Their robustness and reliability across different *k* settings is indicated by consistently achieving lower error metrics (MAE, MSE, RMSE, MAPE) and higher NSE values.

**Table 4:** Framework results of all dataset

		Regression models					Deep learning models			
*k* value	Evaluation metric	Linear regression	Random forest regressor	Decision tree regressor	K-Neighbors regressor	XGBoost regressor	ANN	DNN	CNN	LSTM
*k*= All	MSE	0.0863	0.064	0.0922	0.0864	0.0708	0.0877	0.0801	0.078	0.0807
	MAE	0.2251	0.2078	0.2628	0.2417	0.222	0.2309	0.2212	0.2276	0.2342
	RMSE	0.2937	0.253	0.3036	0.2939	0.2661	0.2961	0.283	0.2793	0.284
	NSE	0.6364	0.7391	0.6101	0.6345	0.7523	0.6768	0.6352	0.6752	0.677
	MAPE (%)	10.228	7.889	12.322	10.409	9.257	9.405	10.038	11.152	12.275
*k* = 9	MSE	0.116	0.0678	0.0767	0.0875	0.0605	0.0742	0.0892	0.0783	0.0764
	MAE	0.2555	0.209	0.2247	0.2577	0.2134	0.2162	0.2369	0.2269	0.2323
	RMSE	0.3406	0.2604	0.277	0.2958	0.246	0.2723	0.2986	0.2798	0.2764
	NSE	0.694	0.7092	0.5695	0.6299	0.7439	0.7027	0.6265	0.6537	0.6867
	MAPE (%)	10.523	8.441	13.693	12.156	8.923	10.769	11.193	10.871	12.103
*k* = 7	MSE	0.1118	0.0726	0.095	0.0958	0.074	0.0867	0.0797	0.0881	0.077
	MAE	0.2528	0.2201	0.2531	0.2679	0.233	0.2452	0.2218	0.2357	0.2304
	RMSE	0.3344	0.2695	0.3082	0.3096	0.272	0.2944	0.2824	0.2968	0.2774
	NSE	0.6911	0.6891	0.598	0.5949	0.687	0.6781	0.6209	0.6441	0.6799
	MAPE (%)	10.674	9.782	11.626	12.611	11.951	11.393	12.57	12.104	11.181
*k* = 5	MSE	0.0734	0.0771	0.1103	0.0933	0.0707	0.0764	0.0841	0.0732	0.0688
	MAE	0.2152	0.2267	0.2773	0.26	0.2233	0.2199	0.2406	0.2185	0.2186
	RMSE	0.2709	0.2777	0.3322	0.3054	0.2659	0.2764	0.29	0.2706	0.2623
	NSE	0.6895	0.6604	0.5332	0.6055	0.7009	0.6915	0.6224	0.6818	0.6907
	MAPE (%)	10.0313	9.683	12.009	11.037	10.22	10.638	11.056	11.536	11.639
*k* = 3	MSE	0.0712	0.0725	0.1103	0.0925	0.0707	0.074	0.0783	0.075	0.0726
	MAE	0.2152	0.2228	0.2773	0.2578	0.2233	0.2162	0.2278	0.2251	0.2174
	RMSE	0.2669	0.2693	0.3322	0.3042	0.2659	0.2721	0.2798	0.2739	0.2694
	NSE	0.6587	0.6583	0.5332	0.6086	0.7009	0.7107	0.6668	0.6941	0.71
	MAPE (%)	10.506	9.399	12.009	11.426	10.22	10.567	10.54	10.148	10.456

Table 5 displays the results of the framework applied on the rainy season dataset. Linear regression model performs well with low MSE and high NSE for certain values of *k*. Random Forest regressor generally shows competitive performance across different values of *k*, especially for *k* = 3 and *k* = 5 based on MSE and NSE. Similar to Random Forest, Decision Tree regressor also exhibits varying performance across different values of *k*. K-Neighbors regressor has varying performance but generally performs well for lower values of *k* (*k* = 3 and *k* = 5) based on MSE and NSE. XGBoost regressor consistently performs well across different values of *k*, particularly for *k* = 3 and *k* = 5. Deep learning models, such as ANN, DNN, CNN, and LSTM, generally outperform traditional regression models in terms of MSE and NSE for different values of *k*, especially for *k* = All and *k* = 9. However, they require higher computational complexity. Lower values of k (such as *k* = 3 and *k* = 5) generally result in better performance across most models, with lower MSE and higher NSE. Higher values of *k* (such as *k* = All and *k* = 9) exhibit more variability in model performance, with certain models performing better than others.

**Table 5:** Framework results of rainy season dataset

		Regression models					Deep learning models			
*k* value	Evaluation metric	Linear regression	Random Forest regressor	Decision Tree regressor	K-Neighbors regressor	XGBoost regressor	ANN	DNN	CNN	LSTM
*k* = All	MSE	298.2598	0.0169	0.0404	0.0355	0.0335	0.0296	0.0212	0.0175	0.0287
	MAE	8.3849	0.1108	0.1467	0.1394	0.1592	0.1488	0.1266	0.1164	0.1454
	RMSE	17.2702	0.1299	0.201	0.1884	0.1831	0.1721	0.1458	0.1325	0.1694
	NSE	0.137	0.9136	0.818	0.8286	0.8381	0.8176	0.8569	0.8798	0.8617
	MAPE (%)	48.402	6.442	11.535	5.711	10.065	12.455	0.765	8.504	9.909
*k* = 9	MSE	5.7041	0.0192	0.0494	0.0106	0.0361	0.0258	0.0349	0.0222	0.0259
	MAE	1.1108	0.1127	0.1631	0.0786	0.165	0.1408	0.1433	0.1235	0.1406
	RMSE	2.3883	0.1387	0.2223	0.1029	0.19	0.1606	0.1869	0.1491	0.1609
	NSE	0.3495	0.9291	0.8049	0.9489	0.8257	0.8982	0.9097	0.8256	0.8764
	MAPE (%)	25.159	6.404	12.402	4.428	9.734	6.576	7.875	10.618	8.828
*k* = 7	MSE	3.0971	0.0191	0.0445	0.0317	0.0281	0.0246	0.0313	0.0186	0.0313
	MAE	0.8295	0.108	0.1631	0.1233	0.1531	0.1332	0.1475	0.119	0.1497
	RMSE	1.7599	0.1384	0.211	0.178	0.1677	0.1569	0.177	0.1365	0.1769
	NSE	0.4308	0.9104	0.8033	0.847	0.8643	0.8357	0.8825	0.8776	0.8712
	MAPE (%)	17.742	6.042	5.695	7.372	9.426	11.181	7.942	6.138	9.322
*k* = 5	MSE	0.4923	0.026	0.0472	0.0321	0.0408	0.0299	0.0249	0.0167	0.0201
	MAE	0.37	0.1259	0.1706	0.1116	0.1867	0.1502	0.1192	0.116	0.1247
	RMSE	0.7017	0.1612	0.2172	0.1792	0.202	0.173	0.1579	0.1292	0.1417
	NSE	0.6326	0.882	0.7723	0.8451	0.8031	0.9022	0.9196	0.9139	0.9103
	MAPE (%)	7.873	8.15	9.681	7.194	11.537	7.345	7.137	6.271	6.787
*k* = 3	MSE	0.0156	0.0224	0.0844	0.0204	0.0536	0.0266	0.0157	0.0178	0.0201
	MAE	0.1037	0.1301	0.2285	0.12	0.1887	0.1402	0.1104	0.1212	0.124
	RMSE	0.1247	0.1496	0.2906	0.1427	0.2315	0.1632	0.1252	0.1334	0.1416
	NSE	0.9249	0.8706	0.6698	0.9017	0.7413	0.9209	0.9297	0.9304	0.9153
	MAPE (%)	7.019	9.195	13.807	7.779	12.361	7.135	6.801	6.378	8.039

The dry season dataset’s framework results are presented in Table 6. Linear regression model performs well with low MSE and high NSE for certain values of *k*. Random Forest regressor generally shows competitive performance across different values of *k*, especially for *k* = 3 and *k* = 5 based on MSE and NSE. Similar to Random Forest, Decision Tree regressor also exhibits varying performance across different values of *k*. K-Neighbors regressor has varying performance but generally performs well for lower values of *k* (*k* = 3 and *k* = 5) based on MSE and NSE. XGBoost regressor consistently performs well across different values of k, particularly for *k* = 3 and *k* = 5. Deep learning models, such as ANN, DNN, CNN, and LSTM, generally outperform traditional regression models in terms of MSE and NSE for different values of *k*, especially for *k* = All and *k* = 9. However, they require higher computational complexity. Lower values of *k* (such as *k* = 3 and *k* = 5) generally result in better performance across most models, with lower MSE and higher NSE. Higher values of *k* (such as *k* = All and *k* = 9) exhibit more variability in model performance, with certain models performing better than others.

**Table 6:** Framework results of dry season dataset

		Regression models					Deep learning models			
*k* value	Evaluation metric	Linear regression	Random Forest regressor	Decision Tree regressor	K-Neighbors regressor	XGBoost Regressor	ANN	DNN	CNN	LSTM
*k* = All	MSE	0.3669	0.0731	0.1372	0.0589	0.0545	0.0728	0.0857	0.0744	0.061
	MAE	0.4687	0.2203	0.3226	0.2284	0.2084	0.2363	0.2393	0.2191	0.2112
	RMSE	0.6057	0.2703	0.3704	0.2426	0.2334	0.2699	0.2928	0.2728	0.2471
	NSE	0.2729	0.7189	0.4442	0.7502	0.7689	0.6714	0.6552	0.7035	0.7556
	MAPE	22.307	10.657	12.532	9.131	10.884	6.059	6.894	6.985	9.682
*k* = 9	MSE	0.4463	0.0811	0.1533	0.0763	0.0804	0.0613	0.0785	0.0791	0.0579
	MAE	0.421	0.2416	0.3194	0.2567	0.2447	0.2261	0.2287	0.2326	0.2085
	RMSE	0.668	0.2848	0.3916	0.2761	0.2836	0.2475	0.2803	0.2813	0.2406
	NSE	0.5842	0.6569	0.348	0.6764	0.6587	0.652	0.6642	0.6761	0.7525
	MAPE	6.324	12.084	18.455	9.672	11.232	6.695	6.252	6.347	9.574
*k* = 7	MSE	0.6131	0.0554	0.0827	0.0656	0.0542	0.0624	0.074	0.0714	0.0575
	MAE	0.4531	0.217	0.2525	0.2313	0.226	0.202	0.2269	0.2168	0.2072
	RMSE	0.783	0.2353	0.2876	0.2561	0.2329	0.2498	0.272	0.2671	0.2398
	NSE	0.0985	0.7768	0.5047	0.7218	0.7698	0.8031	0.7178	0.6591	0.7422
	MAPE	44.38	10.697	6.439	8.918	12.675	12.162	8.004	6.673	9.952
*k* = 5	MSE	0.1493	0.0545	0.0767	0.0314	0.0334	0.0538	0.0509	0.0435	0.0551
	MAE	0.3023	0.2037	0.2127	0.1407	0.1642	0.2169	0.1811	0.1787	0.205
	RMSE	0.3864	0.2335	0.277	0.1771	0.1827	0.232	0.2256	0.2086	0.2348
	NSE	0.6903	0.7755	0.6745	0.8669	0.8584	0.7935	0.8094	0.7975	0.776
	MAPE	7.126	9.117	15.028	3.998	10.139	10.824	6.527	7.558	9.899
*k* = 3	MSE	0.0715	0.063	0.0656	0.0496	0.0468	0.0481	0.0483	0.0419	0.0475
	MAE	0.2266	0.2261	0.2322	0.1915	0.2027	0.1947	0.1894	0.1851	0.1933
	RMSE	0.2674	0.251	0.2562	0.2228	0.2163	0.2194	0.2198	0.2047	0.218
	NSE	0.6966	0.7392	0.7148	0.7894	0.8014	0.8144	0.795	0.8026	0.798
	MAPE	8.54	11.303	10.726	6.959	11.338	12.725	6.68	8.627	10.567

Let’s have a closer look at the technical aspects of the performance of machine learning and deep learning models. Conventional regression models such as Linear regression, Decision Tree regressor, and K-Neighbors regressor demonstrate satisfactory performance, particularly for lower *k* values. They often yield interpretable outcomes and demand less computational complexity compared to deep learning models. Deep learning models such as artificial neural networks (ANN), deep neural networks (DNN), convolutional neural networks (CNN), and long short-term memory (LSTM) networks generally outperform traditional regression models, especially when handling intricate data patterns and larger *k* values. However, they involve higher computational expenses and may require a larger amount of data for training. Both Random Forest and XGBoost regressors demonstrate exceptional performance on a range of datasets and values for *k*. These models are renowned for their capacity to handle intricate data relationships and generate reliable predictions. They maintain impressive performance even when *k* is increased, highlighting their adaptability and efficacy in diverse situations. Decreasing the value of *k* typically leads to improved performance among most models, as evidenced by lower error metrics like MSE and higher values of NSE. This implies that utilizing a smaller number of features for prediction often leads to more precise models, potentially by minimizing noise and overfitting. On the other hand, higher values of *k*, such as incorporating all features (*k* = All) or larger values like *k* = 9, result in greater variability in model performance. In these instances, certain models may outperform others, underscoring the significance of choosing an appropriate *k* value based on the dataset’s characteristics and prediction needs. In various *k* values, LSTM consistently surpasses other deep learning models, especially in situations involving temporal dependencies. Its proficiency in capturing long-term dependencies makes it highly suitable for time-series data, as evidenced by your findings where it outperforms not only traditional regression models, but also other deep learning architectures. Although deep learning models typically demonstrate better performance, they also bring about greater computational complexity and therefore require more computational resources for both training and inference. This trade-off must be taken into account when choosing a model for a specific task, particularly in situations where computational resources are restricted.

## Conclusions

In summary, the growing global water crisis, characterized by deterioration in both water quality and quantity, has raised concerns about the viability of what was once an infinite resource. Groundwater, historically viewed as an unspoilt source, has gained importance due to its comparative purity and resistance to pollution from surface water. This study explores the complexities of groundwater quality.

- Particular attention is paid to the presence of geogenic contaminant: fluoride.
- To identify probable geogenic contamination, we created a prediction model utilizing several regression and deep learning techniques.
- The SelectKbest feature selection method used to uncover influential variables and raise prediction accuracy in our framework.
- We were able to spot changes in groundwater pollution and get a full grasp of its dynamics by investigating seasonal data.
- The assessment methods employed confirm the effectiveness of our predictive algorithms in correctly identifying geogenic pollution.

The experiments revealed important information on the environmental characteristics and factors that contribute to groundwater contamination.

- The entire dataset exhibits a wide range of values, observed in the data distribution analysis, with large outliers for elements such as aluminum (Al), cadmium (Cd), cobalt (Co), and manganese (Mn).
- Tighter distributions and distinct variances were detected in the value ranges of each component in the “rainy season” and “dry season” datasets.
- Trait correlation analysis found substantial linear correlations between the elements, with boron (B) and phosphorus (P) exhibiting a significant positive correlation and chromium (Cr) and copper (Cu) showing a moderate negative correlation.
- The findings of feature selection revealed that boron (B) and cadmium (Cd) had consistent significance across all data sets and play important roles in groundwater contamination.
- In the machine learning framework evaluation, the XGBoost regressor outperformed in modeling both the “all dataset’ and “rainy season,” whereas the convolutional neural network (CNN) outperformed in the “dry season” dataset.

This study offers an extensive comprehension of the dataset’s features, fundamental components in groundwater contamination, and the flexibility of different modeling approaches under varying feature selection scenarios. This study is groundbreaking as previous research has not extensively explored predicting groundwater quality with such a diverse range of techniques, particularly regarding heavy metal parameters. Our discoveries hold significant potential in proactively safeguarding water resources and public health against the increasing risks of geogenic groundwater contamination. Fluoride pollution in groundwater is mostly geogenic. However, if high fluoride content is caused by factors related to the geological structure, it is not possible to reduce it at the source. However, if it is caused by anthropogenic pressure, the best solution is to reduce or eliminate the pollutant source.

Our research suggests that water management authorities should take action to ensure the establishment of a comprehensive monitoring network, especially for fluoride, in groundwater. This can help make plans to prevent further deterioration of the groundwater quality situation, specifically for fluoride and other heavy metals. It will enable local governments to take action to take stronger and tougher measures to ensure safe drinking water. It will also ensure that measures are taken for the sustainable and safe use of groundwater resources. In this context, our recommendations are the local government must provide alternative water sources for drinking purposes or establish a water treatment plant (fluoride removal units) to remove high fluoride. In addition, in future studies, the methods tried for the first time in this study can be used without the need for the existing monitoring system. In other words, new methods such as data mining, deep learning, machine teaching, and artificial intelligence that estimate fluoride without measuring fluoride values by considering toxic parameters should be actively used in the evaluation.

## Data Availability

The datasets used and/or analyzed during the current study are available from the corresponding author on reasonable request.

## Code availability

This work does not contain “software application or custom code.”
